# Genotoxicity in Unconventional Mammalian Models of Wild, Urban, and Agricultural Ecosystems: A Systematic Review Under the One Health Approach

**DOI:** 10.3390/genes16050525

**Published:** 2025-04-29

**Authors:** Nora Bibiana M. Gorla, Mariela Nieves, Daniela Marisol Ferré

**Affiliations:** 1 Laboratorio de Genética, Ambiente y Reproducción (GenAR), Universidad Juan Agustín Maza (UMaza), Mendoza C5519, Argentina; dferre@profesores.umaza.edu.ar; 2 Consejo Nacional de Investigaciones Científicas y Técnicas (CONICET), Godoy Cruz C2290, Argentina; 3 Grupo de Estudios en Arquitectura Genómica de Mamíferos (arGENma), Dirección de Investigaciones Centro de Educación Médica e Investigaciones Clínicas “Norberto Quirno” (CEMIC-CONICET), Ciudad Autónoma de Buenos Aires (CABA) C1431, Argentina; mnieves@cemic.edu.ar

**Keywords:** agro ecosystems, cattle, wild scenarios, primates, homelike environment, dogs, contaminants, genetic toxicology, One Health

## Abstract

**Background/Objectives:** This systematic review evaluates unconventional mammalian models from wild, agricultural, and urban/domestic ecosystems for genotoxicity assessment under the One Health framework. Non-human primates (NHPs), cattle, and domestic dogs are analyzed as sentinel species due to their distinct environmental niches, unique human interactions, and species-specific traits. In conjunction with this, evidence is presented about the in vitro use of cells of these mammals for the genotoxicological evaluation of different chemical substances, such as veterinary drugs, environmental pollutants, and pesticides. The synthesis focuses on standardized genetic toxicology assays (e.g., chromosomal aberrations, micronucleus, comet assay) aligned with Organization for Economic Cooperation and Development (OECD) guidelines. **Methods:** A structured search of international literature identified studies employing OECD-compliant genotoxicity assays in NHPs, cattle, dogs, and others not listed in OECD. Data was categorized by species, assay type, chemical class evaluated, environmental context (wild, agricultural, urban), and merits of the papers. **Results:** NHPs, despite their phylogenetic proximity to humans, show limited genotoxicity data in contrast to biomedical research, which has been constrained by ethical concerns and fieldwork logistics. Cattle emerge as robust models in agricultural settings due to the abundance of studies on the genotoxic capacity of pesticides, veterinary drug, and environmental biomonitoring, with direct implications for food safety. Domestic dogs are recognized as powerful sentinels for human health due to shared exposomes, physiological similarities (e.g., shorter cancer latency), and reduced lifestyle confounders; however, genotoxicity studies in dogs remain sparse compared to chemical exposure monitoring or cancer research. **Conclusions:** This review advocates for expanded, integrated use of these models to address genotoxic threats across ecosystems, which would benefit both animal and human health. In the application of biomonitoring studies with sentinel animals, a critical gap persists: the frequent lack of integration between xenobiotic quantification in environmental and biological samples, along with genotoxicity biomarkers evaluation in sentinel populations, which hinders comprehensive environmental risk assessment.

## 1. Introduction

Numerous scientific studies have examined the use of different genotoxicity tests, in exposed human groups and in conventional laboratory biological systems: traditionally bacteria (*Salmonella typhimurium*), rodents, *Allium cepa*, or cell lines. An even smaller number of studies have been conducted on non-conventional animals.

As an alternative to the use of conventional biological systems in genotoxicity testing, the use of mammals in their natural environment as sentinel animals for public and environmental health is proposed as a genuine alternative. Under the integrative concept of “One health”, in which “the health of humans, domestic and wild animals, plants, and the wider environment (including ecosystems) are closely linked and interdependent” (World Health Organization (WHO): https://www.who.int/health-topics/one-health#tab=tab_1, accessed on 15 January 2025) the objective of this paper was to review, analyze and synthesize the existing literature on the use of non-human primates (NHPs), cattle, and companion canines to assess the genotoxicity of xenobiotics that may be used or present in their usual environments. These three species, coming respectively from very different environments: wild, agricultural, and residential, have their own qualities that make them attractive representatives to evaluate the adverse effects on genomic integrity due to environmental contaminants. These animal models would enable addressing real-world challenges associated with adverse effects caused by mutagenic or genotoxic agents, offering a much greater certainty in assessing scenarios and broader potential for applying solutions—both pharmacological interventions in individuals and remedial measures in ecosystems—compared to conventional biological models.

Xenobiotics of living beings in general, with genotoxic and carcinogenic potential, are classified in physical, biological and chemical agents. In this review, the studies being analyzed are primarily from short-term assays for detecting mutagens and carcinogens conducted with chemical ones.

### 1.1. Mutagenesis and Genotoxicity Are Part of the Development of Cancer and Other Diseases

At this point in modernity, it is imperative to evaluate the toxicity, including genotoxicity, of an unprecedented number of newly synthesized chemical substances, as well as to review those that are already marketed. This process includes an assessment of carcinogenic risk based on animal genotoxicity studies and short-term mutagenicity assays [1,2,3].

Genotoxicity involves agents damaging DNA or cellular targets, potentially causing mutations (mutagenicity) at the DNA base level (point mutations, adducts) or chromosomal level (breaks leading to rearrangements, aneuploidy) [2]. Somatic cell mutations are linked to cancer risk, while germ cell mutations can cause hereditary diseases. Genotoxicity, mutagenicity, clastogenicity, and aneugenicity are critical processes in genetic toxicology assays, as they can indicate a risk of carcinogenesis and genetic diseases [1,4]. Clastogenicity refers to the ability of an agent to produce breaks in chromosomes, while aneugenicity refers to its ability to induce aneuploidies. Permanent changes occur in the sequence of DNA bases in the form of mutations or aberrations can also interfere with DNA transcription, replication, trigger cell death or cellular senescence, thus contributing to wider, and more complex process of aging [5].

For genotoxic substances that interact with DNA—either directly or indirectly after a metabolic transformation—it is commonly assumed that there is no threshold in their mechanism of action; that is, all doses have a potential effect. Threshold-based mechanisms are conceivable for genotoxic agents that do not react with DNA, such as those that affect the function and organization of the mitotic spindle inducing aneuploidy [6], or that affect chromosomal integrity through inhibition of topoisomerase [7], cause DNA damage indirectly through oxidative stress [8], affect genes responsible for DNA repair, immune genes, and others regulating the cell cycle and apoptosis [9].

Genetic toxicity or genotoxicity is significantly different than other types of toxicity: at low concentrations of an adverse genotoxic agent, genetic alterations can lead to chronic toxic effects that only manifest after long periods after exposure.

DNA damage accumulation in somatic cells links to cancer and degenerative conditions like accelerated aging [10], immune dysfunction [11], and cardiovascular [12] and neurodegenerative diseases [13], while germ cell damage affects reproduction and offspring health via mutations or hereditary diseases. Approximately 90% of identified carcinogens are also mutagens [14].

Biomarkers are quantitative measures of changes in a biological system with respect to its normal state in response to exposure to pollutants, at the molecular, cellular, or physiological level. In genotoxicity tests applied in population biomonitoring, different biomarkers are evaluated and quantified [15].

Genomic instability is a distinctive feature of cancer, and the analysis of DNA damage at the chromosomal level is a crucial part in the study of genotoxicity and the process of carcinogenesis. Chromosomal instability (CIN), the most common level of genomic instability in cancers, is defined as the rate of loss or gain of chromosomes through successive divisions; all of this determines that the DNA of cancer cells is highly unstable [16]. CIN can be evaluated in terms of numerical chromosomal aberrations (CA) and structural aberrations such as single-strand breaks (ssb) or double-strand breaks (DNA-dsb), micronuclei (MN), nucleoplasmic bridges (NPB), and nuclear buds (NBUD).

Genotoxicity assessment tests to analyze the genotoxic capacity of a chemical or to be applied in population biomonitoring to determine the level of CIN should be performed following the guidelines of standardized protocols, such as those of the Organization for Economic Cooperation and Development (OECD).

### 1.2. Test Guidelines for Genetic Toxicology (OECD)

Two types of genetic toxicology studies are considered, which will be reviewed here: (1) those that measure mutagenicity, or direct and irreversible damage to DNA, and (2) those that measure genotoxicity [17].

The OECD guidelines include the 13 Test Guideline for Genetic Toxicology (TG) for testing genotoxicity that help to assess the effects of chemical substances on human health [17]. The bacterial test TG 471: in vitro bacterial reverse mutation assay (Ames test), and the TG 490: in vitro gene mutation assays using the thymidine kinase (TK) gene [18] evaluate gene mutation. The third assay that evaluates in vitro gene mutation is TG 476: in vitro mammalian cell gene mutation tests using the Hprt or xprt genes, hypoxanthine-guanine phosphoribosyl transferase or xanthine-guanine phosphoribosyl transferase [19]. Continuing with in vitro tests, two tests are suggested that evaluate chromosomal aberrations; TG 473: in vitro mammalian chromosomal aberration test [20] and TG 487: in vitro mammalian cell micronucleus test [21].

In addition to the in vitro tests, there are six in vivo assays, one of them with gene mutation as final target: TG 488: Transgenic rodent somatic and germ cell gene mutation assays [22]. On the other hand, five assays with CA as final target: two assays designed for somatic cells TG 474: Mammalian erythrocyte micronucleus (MN) test [23], and TG 475: Mammalian bone marrow chromosomal aberration test [24]; and others targeted to germ cells TG 478: Rodent dominant lethal test [25], TG 483: Mammalian spermatogonia chromosomal aberration test [26], and TG 485: Mouse heritable translocation [27].

Finally, two assays assess primary DNA damage as the ultimate target, TG 486: Unscheduled DNA synthesis (UDS) test with mammalian liver cells in vivo [28]; and TG 489 in vivo mammalian alkaline comet assay [29].

Despite this imbalance, nine in vivo tests and only four in vitro tests, most regulatory authorities have increased their commitment to avoid the unnecessary use of animals in toxicological testing and require three tests: a bacterial gene mutation test, an in vitro test in mammalian cells that detects gene mutations and/or CA, and an in vivo test to assess chromosomal damage. If genotoxicity is identified, it is advisable to perform an assay involving evaluation in gonads. If the latter is positive, a quantitative risk assessment in rodents may be required [30]. The detection of genetic damage that can occur in germ cells and be transmitted to offspring protocoled in the OECD for that purpose is: Dominant lethal and the in vivo Transgenic Rodent Somatic and Germ Cell [31].

Two tests can determine whether a chemical can cause chromosomal damage and/or aneuploidy: MN and CA. Many studies have shown that substances that can induce CA and MN are also capable of inducing transmissible chromosomal mutations, such as reciprocal translocations, stable translocations and aneuploidy [31]. The buccal MN test has been used in numerous biomonitoring human populations exposed to potential carcinogens [32]. Recently, the MN assay in oral mucosa epithelial cells has been adapted for bovine [33] and canines (Ferré, pers. comm).

It is important to clarify that OECD TGs have been designed to evaluate the potential genotoxicity of chemical substances (in vitro and in vivo). Nonetheless, the tests have also been and are used in biomonitoring, to study animals in their natural habitats in situ, obtaining biological samples and then perform cellular cultures in vitro, in an ex vivo approach. Hence, these animals are identified in the literature as sentinels or bioindicators of genotoxic agents potentially present in the environment [34,35,36,37].

The assessment of the genotoxic potential of a chemical is one of the first steps in the risk assessment process of a substance [38]. It has also been recommended that in vitro tests should be followed by conducted the smallest number of in vivo tests possible while maintaining scientific accuracy [39] due to ethical concerns.

## 2. Non-Conventional Mammals and Their Use in Genotoxicity Assays

Moving away from the use of conventional laboratory animals, primarily rodents, and employing non-conventional species requires “thinking outside the box”. This approach offers an opportunity to evaluate adverse effects on genetic material in diverse genomes under more realistic conditions. Additionally, it enables comparative genomic studies, which are a fundamental contribution to the development of innovative experimental designs for testing the genotoxic capacity of various agents across different animal groups. This approach opens the possibility of not intentionally intoxicating animals but instead using them as part of environmental biovigilance in their natural habitats. It is from this “One health” perspective that the present review can offer an added value to the knowledge of the genotoxicity of chemical compounds produced in the current lifestyle.

### 2.1. Wildlife Non-Human Primates and Its Relation to Human and Ecosystem Health

The Order Primates includes non-human primates and humans. Taxonomically, it is divided into Strepsirrhini (lemurs, galagos, and lorises) and Haplorrhini (tarsiers, monkeys, Old World Monkeys (OWM) and New World Monkeys (NWM), gibbons, and great apes (orangutan, gorilla, chimpanzee, and humans)). NHPs weigh between approximately 30 g (Berthe’s mouse lemur) and 250 kg (eastern lowland gorilla) [40]. They are characterized by a large brain relative to other mammals, a feature especially noticeable in great apes. They exhibit sexual dimorphism, including body mass, canine size, and coloration. Primates are among the most social animals, forming pairs or family groups, single-male harems and multi-male/multi-female groups.

NHPs are distributed in the tropical regions of South and Central America, Africa, and Asia. They play key roles in tropical ecosystems while providing valuable insights into human biology, behavior and evolution [41].

As human-wildlife animal interactions have increased, the study of human–wildlife interactions and conflicts has established itself as an important and continuously growing area of concern. The relevance of the topic is reflected in the advent of relatively new approaches, such as Human–Animal Studies (HAS) and Human–Wildlife Conflict (HWC) [42]. Viewing ourselves as an integral part of a complex, constantly interacting ecosystem allows us to address the myriad ways in which our lives intersect and are influenced by the lives and deaths of other species. However, these complex intersections often lead to conflicts between humans and wildlife with high costs for both. For humans this includes livestock and crop losses or property damage. For wildlife, punitive deaths often result at the individual level; at the population level, during population decline, long-term survival of species is threatened [43].

At the same time, close interactions between humans and NHPs can result into transmission of zoonotic diseases, especially viral diseases such as herpes, measles, Ebola, rabies, and hepatitis. In this area, NHPs are used in research around the world because of their physiological similarity to humans.

The “One World, One Health” concept [44] emphasizes the interconnectedness of human, domestic animal, and wildlife health and underscores the value of studying wildlife in human-modified environments. This approach helps assess ecosystem viability, understand disease threats impacting populations and food supplies, and gauge the effects of environmental pollutants on long-term survival and biodiversity.

While this review focuses essentially on chemical agents and their genotoxicity, it is important to note that emerging zoonotic diseases pose a serious threat to both human and veterinary public health and the conservation of wild biodiversity [44], and that a large number of studies conducted with NHPs have been conducted with infectious agents [45]. The monitoring of diseases in wild species has made it possible to explore increases in the incidence of metabolic, endocrine, reproductive, or infectious diseases. Primates are considered excellent epidemiological surveillance sentinels for zoonotic diseases, such as yellow fever, malaria, and schistosomiasis [46,47,48].

Also, although humans have always shared habitats with NHPs, the dynamics of interactions between humans and NHPs have changed dramatically in the recent past. Therefore, transmission of zoonotic pathogens is considered the primary threat to nonhuman primate survival and public health in the same landscapes, as increased spatial proximity between species increases the risk of pathogen transmission [49]. In this regard, exposure to potentially harmful chemical agents constitutes a new threat, and NHPs are increasingly approaching cultivated areas where pesticides are used, and contact with chemical contaminants, previously non-existent, are now occurring. Human activities, including logging, land clearing, and agriculture, have led to NHPs increasingly sharing anthropogenically modified landscapes with humans and livestock [50].

To ensure the survival of wild diversity, biomarker monitoring in different animal models can provide important information on the health of a natural environment [51,52,53]. NHPs are a potential candidate for this type of studies, not only because of their high physiological similarity, but because of their similarity in neurobiology and in susceptibility to infectious and metabolic diseases.

In pharmacology and toxicological research, primates like macaques (*Macaca* spp.) and marmosets (*Callithrix* spp.) are commonly used. To examine neurodegenerative diseases such as Alzheimer’s, arteriosclerosis and diseases caused by viral agents, protozoa or bacteria, the biomedical industry frequently utilizes baboons (*Papio* spp.), the vervet monkey (*Chlorocebus aethiops*), African green monkey *(C. pygerythrus*) (Old World Monkeys); squirrel monkey (*Saimiri* sp.), tamarin monkeys (*Saguinus* sp.) and capuchin monkeys (*Cebus* sp. and *Sapajus* sp.) (New World Monkeys) [54]. Capuchin monkeys are natural reservoirs of *Trypanosoma cruzi*, making them an ideal model for the study of Chagas disease [55]. Baboons, on the other hand, are widely used in reproductive biology studies [56].

The exposure of the common marmoset (*Callithrix jacchus*) to Fe and Cr in the wild was investigated in order to evaluate the exposure of these species to chemical agents. For this purpose, the presence of the metals was detected and measured in liver, hair, and bones of animals in the wild [57]. The choice of this species as a potential model for environmental biomonitoring is based on the previous knowledge that *C. jacchus* is a species adaptable to different environments, with a wide geographical distribution. Furthermore, it inhabits urban and peri-urban forests close to human occupations and has a varied diet.

### 2.2. Evaluating the Genotoxicity in Wildlife Species: The Non-Human Primates

An exhaustive search was performed in the Google Scholar search engine using specific keywords: genotoxicity study; non-human primates; micronucleus assay; chromosome aberration assay: sister chromatid assay; imidazoles; antiparasitic drugs. The papers obtained were reviewed according to the following criteria: (1) that a specific approach such as in vitro, in vivo, and/or ex vivo on the study of the genotoxic potential of pesticides and drugs used in human diseases had been described in detail; (2) that biomarkers commonly used in genotoxicity studies had been applied for the analysis of DNA damage; and (3) that they were not mere pathological descriptions at the organ level in response to exposure. In NHPs, approximately 35 studies from the last 40 years were obtained using the pre-established keywords. Only 16 papers (presented in Table 1) met the pre-established criteria.

Throughout the life of an organism, the genome is in constant interaction with various agents that can affect its integrity, which, if negatively impacted, could translate into genomic instability. Likewise, the complexity and diversity in the spatial and dynamic organization of chromatin determines varying degrees of stability, susceptibility and, therefore, response to possible damage.

Comparative NHP genotoxicity studies offer insights into testing methods and genomic dynamics. Research, which has been evolving since the 1980s in fragile sites [68,72], now investigates genomic instability (e.g., SCEs, Cell Proliferation Kinetics (CPC)) and differential responses to in vitro chemical exposures (e.g., antiparasitics used in human and veterinary medicine) across various species [65,66], particularly Neotropical primates like *Sapajus* spp. [67]. These efforts aim to establish these NHPs as valuable research models according to the principles of humane experimental [73], by characterizing their unique genomic sensitivities and responses. The genomic sensitivity was analyzed in four species of NWM with different genomic characteristics in order to compare the response of each individual genome, characterizing the spontaneous occurrence of SCEs. Among them, in *S. cay*, a low proportion of statistically significant unstable bands was observed, suggesting a relatively stable genome and the existence of some type of protection against endogenous damage [66].

Recent studies in *S. cay* have documented dicentric chromosome-like figures following in vitro exposure of peripheral blood lymphocytes to bleomycin, a radiomimetic agent, at concentrations below those typically used in human studies [74]. Notably, while human peripheral blood lymphocyte cultures predominantly exhibit lagging chromosomes or anaphase bridges—common indicators of chromosomal instability—the observed differences suggest potential interspecies variation in bleomycin’s mechanism of action between primates. The micronucleus assay further corroborated these findings, revealing not only significant micronuclei induction but also intercellular bridges that were found exclusively in exposed binucleated cells [74,75].

Analysis of NHP genetic toxicology research over approximately 30 years reveals a significant shift. The first thing that stands out is that the focus, the varying objectives and the needs for knowledge generation have been in flux; however, basically the same species of NHP previously mentioned are still being used. Early studies were often invasive in vivo experiments involving post-sacrifice analysis. Newer research predominantly employs in vitro methods (cell/lymphocyte cultures) using fewer animals, allowing broader test batteries and better alignment with animal welfare considerations, though both approaches have overlapped chronologically.

A third point that emerges from analyzing Table 1 is that, over the last 15 years, in regard to the studies done on New World Monkeys, the focus has shifted from the genome damage caused by potentially harmful agent exposure to the analysis of genomic instability and the response of different genomes. Thus, studying the dynamic responses of genomes clarifies the mechanistic aspects of the response to endogenous or exogenous cellular stimuli and allows evaluation models to be obtained that reveal the biological consequences of the exposure to agents of therapeutic or environmental interest. Other studies on the effects at the level of genomic organization based on the dynamic-structure relationship are scarce, and it is for this reason that the use of agents with known clastogenic action is highly useful, as they could allow genomic responses that have not been described thoroughly until now to be elucidated.

Working with NHPs in captivity (zoological gardens, breeding centers, rehabilitation centers, and vivariums) presents practical advantages regarding the type of research projects that can be undertaken [76,77]. Undoubtedly, a project that involves capturing, handling, sedating, extracting peripheral blood, or conducting biopsies is likely to be more feasible and less stressful for the animals when conducted with captive primates rather than wild ones. The same studies, conducted in the field with free animals, typically enter in conflict with studies on ecology or behavior of primates because any handling involves waiting at least a week afterward to find the study specimens again. Vivarium, traditionally composed of large colonies of captive-born specimens with complete pedigree and normally without chronic diseases or relevant pathologies for genetic studies, allow for the design of genotoxicity projects. All variables present in free conditions that could hinder, mask, or reduce reliability of the data obtained are better controlled in captivity [74,78,79].

**Figure 1:**
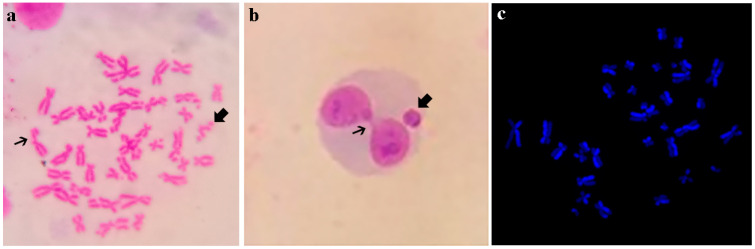
(**a**). Chromosome break (thick arrow) and chromatid break (fine arrow) in *M. fascicularis* lymphocytes exposed in vitro to bleomycin, Giemsa stained (Nieves, pers. comm.). (**b**). Binucleated lymphocyte with micronucleus (thick arrow) and bridge (fine arrow) in in vitro *S. cay* cell micronucleus test, Giemsa Stained [74,75]. (**c**). in vitro SCE in *Ateles chamek* lymphocytes, DAPI stained [65].

The following are photographs illustrating new world monkey chromosomes and biomarkers of genotoxicity used in their study (Figure 1).

Non-human primates are valuable for biomedical research but pose significant ethical challenges. Public concern centers on their cognitive similarity to humans, sparking debates about granting them legal “non-human person” status [80]. The 3Rs Principles advocates minimizing or even rejecting the NHPs use [81,82]; however, their role proves vital in studies requiring high genetic homology, such as SARS-CoV-2 research, T-cell therapies, or Parkinson’s disease models [83,84,85].

In addition to the ethical problems, field-based genotoxicity studies on wild NHPs face logistical hurdles as well: sedation risks, environmental variables, and bidirectional pathogen transmission. Captive research, while improving welfare standards, remains contentious despite advances in habitat quality and care ethics [54].

While traditional zoos and vivariums face decline, NHP use is paradoxically increasing in specialized biomedical research. Captive NHPs are now crucial for karyotypic, evolutionary, and genotoxicity studies, bridging comparative genomics with human health under the “One Health” framework [86,87,88]. This highlights their data’s value but necessitates robust ethical frameworks ensuring interspecies.

In the agricultural environment, information is obtained from another group of animals; the information obtained from applying genotoxicity tests using animals exposed in vivo provides information not only about animal and environmental health but also about the quality of the food consumed by people. The cattle studies discussed below have a unique feature: the information obtained has a practical application to the preventive health of human populations.

### 2.3. Evaluating the Genotoxicity in Agro Ecosystems: The Cattle

Cattle have been the subject of study in genetic toxicology for at least two well-documented reasons in the literature. One is that their cells, which are easily cultivable, constitute suitable biological models for evaluating the genotoxic capacity of various chemical substances in vitro. The other reason is that they are herbivores with biological and behavioral characteristics that inhabit a limited geographic site for months or years; this allows for an evaluation of the effects of chronic exposure to xenobiotic contaminants in the environment that can produce gradual changes in their genome. Cattle are positioned as a sentinel or bioindicator species in the presence of agents with genotoxic capacity in the agricultural ecosystem.

There is abundant scientific evidence in genetic toxicology related to these purposes that link cattle to humans and the environment. They are connected to human health because they are food-producing animals, and to achieve food safety, genotoxicological evaluations in vitro of the chemicals used in the production process are recommended, as they can remain as residues. They are linked to the environment because in vivo and ex vivo genotoxicological assays with cattle allow us to understand the in situ quality of the environment in relation to the presence of agents with genotoxic potential.

#### 2.3.1. Characteristics of the Agricultural Environment and the Meat Production System

The beef cattle value chain begins with primary production (breeding, calf rearing, rearing, and fattening), followed by slaughter and marketing. Key components include animals (calves, heifers, cows, bulls), nutrition, health, management (breeding services, soil/pasture management), and regional environmental factors. They are exposed to introduce the factors or components present in the bovine meat production establishments that can convey a risk for the health of the animals or their food safety products.

Cattle breeding programs utilize genetic selection, natural mating or artificial in-semination, and specific heifer replacement strategies to optimize production efficiency, particularly concerning nutrition costs (cow maintenance, development of pregnancy, lactation, and calf growth) and pasture use. Selection strategies must be applied for the replacement of breeding stock with heifers either from the establishment or acquired in other ways [89,90,91]. There is abundant information about the karyotypic characterization of cattle and the advantages of their use in breeding herds and systems to achieve reproductive efficiencies [92,93,94].

Stocker-backgrounding focuses on animal growth (weight gain), influenced by nutrition, health, biotype, sex, and climate. Systems range from extensive (native grazing) to intensive (confined, formulated rations) or mixed approaches [90].

Fattening or finishing is the next activity in the beef value chain following rearing. Fattening involves high-energy diets (pasture, grains) to promote marbling and fat deposition [90]. Fattening is usually done in confined systems or feedlots, and the diet, mainly based on concentrated feed, and sanitary management are factors that stand out in the productive environment. Both extensive (pasture-based) and intensive production systems, as well as feedlots, are suitable for applying genotoxicological assays; extensive production systems would be more appropriate for environmental quality studies, while intensive systems would be optimal for evaluating the genotoxic activity of veterinary medications.

The sanitary management carried out on animals at each stage of the production chain influences their health status and, for the purposes of this review, the critical points for genotoxicological evaluations that should or could be performed on these animals. Each activity has different sanitary requirements, but these interventions often involve the use of veterinary medications, including vaccines, antimicrobials, and antiparasitics, as well as growth promoters. Proper sanitary management also includes controlling risk factors for diseases such as immunosuppression caused by stress from dehydration, controlling extreme temperatures (high and low), suspended particles, ammonia gases, overcrowding or high animal density, and herding animals of different ages, among others. Veterinary medications have become integral components of the agricultural environment.

#### 2.3.2. Genotoxicity In Vitro Studies of Xenobiotics in Bovine Somatic Cells

Three specific approaches were selected for analysis of genotoxicity studies in bovines: (1) in vitro studies on the effects of xenobiotics on bovine somatic cells; (2) in vitro and in vivo studies conducted over the past 20 years on the genotoxic potential of pesticides and veterinary drugs in bovine cells; and (3) in vivo and ex vivo studies from the last two decades assessing genetic damage in cattle-inhabited contaminated areas. Studies on genotoxicity testing in germ cells and embryos were not included in this review. In the analysis of the available literature on in vitro genotoxicity assays in bovine somatic cells, it is noted that substances and chemical environmental contaminants have been studied for several decades, and veterinary medications used in livestock have been studied more recently.

Numerous in vitro studies using bovine lymphocytes or epithelial cells have assessed the genotoxicity of various chemical environmental contaminants. Examples include mycotoxins (e.g., fumonisin B1, zearalenone and ochratoxin A) [95,96], industrial chemicals e.g., dimethyl phthalate [97], benzene, and bisphenol A [98] and meat-cooking byproducts (heterocyclic amines) [99], utilizing assays like CBMN assay with metabolic activation of lymphocytes using S9 [100,101] and cell lines [102], SCE, CA, comet assay, and fluorescence in situ hybridization (FISH) using whole chromosome painting probes (WCP) [101,102]. The results obtained demonstrate the viability of the animal model for this evaluation.

**Figure 2:**
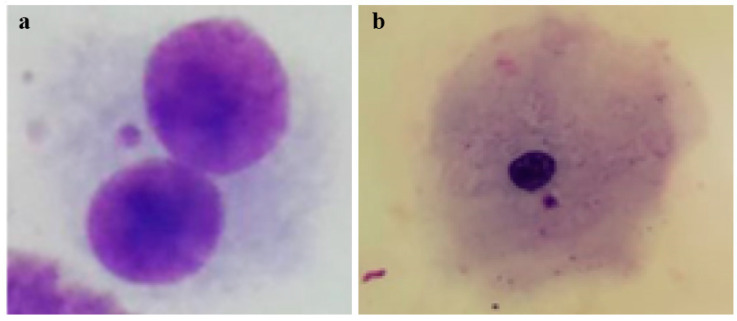
(**a**). Binucleated cell with micronucleus in in vitro Bovine micronucleus test in lymphocytes, Giemsa stained [103]; (**b**). Micronucleus in bovine buccal epithelium cell, Giemsa stained [33].

Figure 2 shows bovine cells used in the in vitro MN assay and bovine buccal epithelial cells used in vivo in the MN assay.

#### 2.3.3. Genotoxicity Studies in Bovine Cells of Chemicals Used in Food Production

Approximately 65 genotoxicological studies in cattle were reviewed, including in vitro and biomonitoring studies. In total, 48 studies met the pre-established selection criteria: studies evaluating the genotoxic potential of environmental xenobiotics on somatic cells; studies conducted in the last 20 years on pesticides and veterinary drugs in bovine cells; and in cattle living in contaminated areas (biomonitoring). The analysis of these studies is presented in Table 2.

Animal-based foods result from interactions among the environment, animals, and humans. From a “One Health” perspective, conditions that promote animal and environmental health support the production of safe food for human consumption. Achieving this goal is challenging, as residues from veterinary medications and other chemicals in food pose a global public health concern. The Pan American Health Organization emphasizes the need to identify environmental health factors linked to food production and consumption [129].

In this context, among various control and prevention strategies, policies promoting Good Livestock and Agricultural Practices stand out to reduce the improper and excessive use of pesticides and veterinary medications in primary production. Surveillance programs for chemical residues in edible animal tissues, fruits, and vegetables are also highlighted to alert about non-compliance with the maximum residue limits (MRLs) adopted by each country or suggested by the Codex Alimentarius. This presents MRLs for veterinary drugs in edible tissues (muscle, liver, kidney, fat) and milk [130]. Finally, guidelines from entities responsible for ensuring food safety and the safety of chemicals involved in food production are emphasized. Among these, and within the framework of this review, the International Cooperation on Harmonisation of Technical Requirements for Registration of Veterinary Medicinal Products (VICH) gains relevance by recommending the evaluation of the genotoxic capacity of veterinary drugs used in the production of food-producing animals for the population [131].

Concern over pesticide and veterinary drug residues in food is high because diet is the main human exposure route, with links to cancer and reproductive disorders. Risk assessment differentiates between aggregate exposure (one substance, multiple routes/pathways) and cumulative exposure (multiple substances, similar mechanisms or modes of action), as defined by bodies like the European Food Safety Authority EFSA [132,133].

The criteria for prioritizing the study of certain chemicals in risk assessments can vary. Genotoxicological studies of pesticides and veterinary drugs are part of the hazard characterization in a risk assessment. In the case of combined risk assessment, EFSA has proposed identifying substances that generate aggregated or cumulative exposures. Following this criterion, Table 2 presents a synthesis of the last 20 years of evaluations of the genotoxic potential of pesticides and veterinary drugs conducted in bovine somatic cells.

From the analysis of the table, there is a clear predominance of studies evaluating the genotoxic potential of pesticides used in agricultural activities, among which herbicides (glyphosate), fungicides (epoxiconazole, fenpropimorph, prothioconazole, tebuconazole, tolylfluanid), and insecticides (thiacloprid, cypermethrin, chlorpyrifos, acetamiprid, bendiocarbamate) stand out as particularly damaging. To a lesser extent, chemotherapeutic veterinary drugs, vaccines, antibiotics, and antiparasitics like mitomycin C, recombinant BCG vaccine, enrofloxacin, doramectin, cypermethrin, and chlorpyrifos [103,106,122,123,125,127] have been studied. Despite the dearth of studies relating to this, it is encouraging to note that these studies have been conducted in recent years, so the trend in evaluating them could be increasing. In the same line, but considering the studies of mixtures of active principles (epoxiconazole + fenpropimorph, prothioconazole + tebu-conazole, chlorpyrifos + cypermethrin), it is evident that those studies of mixtures of veterinary drugs correspond to more recent research [122].

#### 2.3.4. Genotoxicity Studies in Bovines Used for Environmental Biomonitoring

In addition to their use for evaluating the genotoxic capacity of chemical substances, OECD assays have been implemented in cattle for environmental monitoring. Through applications in different scenarios, it has been possible to confirm that this species is suitable as a bioindicator of the presence of agents capable of inducing genotoxic effects. In the most comprehensive of scenarios, genotoxicological assays have been implemented in cattle populations alongside the analytical determination of toxins/contaminants in biological samples such as blood or milk, or in pastures, feed, and water.

It has been demonstrated that the Mammalian Erythrocyte Micronucleus biomarker is sensitive in peripheral blood of cattle studied in Italy and Mongolia [134,135], and it was implemented to evaluate the level of genetic damage in Latvian Brown cows that lived near the Skrunda Radio Location Station (Latvia) and were exposed to electromagnetic radiation [136]. The extent of genetic damage was also thoroughly observed and documented in Japanese Black cows that grazed 12 km from the Fukushima Daiichi Nuclear Power Station (Japan) [137].

The analysis of DNA damage through the comet assay was used in Argentine cattle with hypocupremia, where an association was found between copper deficiency and genetic damage in lymphocytes [138]. It has been implemented in blood cells of cows in Japan exposed to nuclear radiation [137], and in animals *Bos taurus* or *B. taurus* crossed with *Bos indicus* that inhabited lands with agricultural activity within a 18 km radius of two industrial facilities in the United States [139].

CA, CBMN, and SCE assays are the most frequently used tools in cattle biomonitoring studies worldwide. They have successfully evaluated genetic damage linked to environmental factors like arsenic-contaminated water (from two villages namely Gotra and Ramakrishna under Chakda Block of Nadia district in West Bengal, India) [140], dioxins, dioxin-like polychlorodibenzofurans, and dioxin-like polychlorobiphenyls, whose presence was confirmed in milk (Susa Valley, Piedmont, northern Italy) [141], radiation levels proximity to nuclear plants (Wolsong, Uljin and Yeonggwang, Korea) [142], and exposure to reused industrial/textile wastewater (Dladla and Boukallou near Settat, Morocco) [143].

Recently, a study has been presented proposing the use of the Buccal Micronucleus Cytome Assay in cattle. Its simple and cost-effective implementation would facilitate the study of large animal populations inhabiting areas presumed to be contaminated [34].

It is interesting to note that biomonitoring has been conducted in agricultural environments of countries that significantly contribute to beef cattle production for food, but not in all of them. Beef cattle production is led by the United States, Brazil, China, the European Union, India, Argentina, and with less than 5% participation, Australia, Mexico, Russia, among other countries. Meanwhile, the main producers of dairy cattle are the EU, USA, India, China, Russia, Brazil, New Zealand, United Kingdom, Mexico, Argentina, Canada, among other producing countries with less than 10 million tons [144]. In agricultural settings, the interaction between environmental conditions and animals plays a critical role in food safety and security. Therefore, biomonitoring is recommended in regions where primary production of animals for human consumption takes place.

In livestock herds, cytogenetic studies, including genotoxicological ones, are widely used with the objective of optimizing breeding strategies for the herd [92,145,146,147] and to monitor the state of biodiversity, which means having information on the genetic characterization of breeds present exclusively in certain regions to contribute to their conservation [148].

Lastly, it is noteworthy that the interaction between humans and animals is becoming increasingly strong, which is particularly evident for companion animals. Studies indicate that they can significantly improve people’s physical health and psychological well-being. However, by sharing the same environment, air, soil, water, and sometimes food, dogs can play an irreplaceable role in alerting their owners to risks that may affect them, as is shown in the following section.

### 2.4. Evaluating the Genotoxicity in Urban Life: The Dogs

The search for published articles on genotoxicity in dogs was carried out in Google Scholar, without limitation of dates, using the following keywords: chromosomal aberrations, micronuclei, comet, genotoxicity, sister chromatid exchange, genetic instability, chromosomal instability, combined once with dog or with canines, in English and Spanish.

Domestic dogs serve as excellent translational models for cancer research due to striking similarities with human cancers regarding the latency period, clinical signs, metastatic behavior, pathobiology (e.g., tumor heterogeneity, microenvironment), genomic instability, chemoresistance, and multifactorial causes including genetics and environmental factors [149,150]. These aspects have highlighted the enormous potential value of scientific research conducted on spontaneous cancers in dogs to advance our understanding of cancer biology. In this review, the examination of chromosomal and genomic instability assays in dogs and their potential induction by environmental risk factors, particularly contaminants to which humans and dogs are exposed in daily life, is proposed under the hypothesis already advanced by several authors that dogs can act as sentinels alerting humans to the effects of carcinogens in the domestic environment [34,35,151,152].

**Figure 3:**
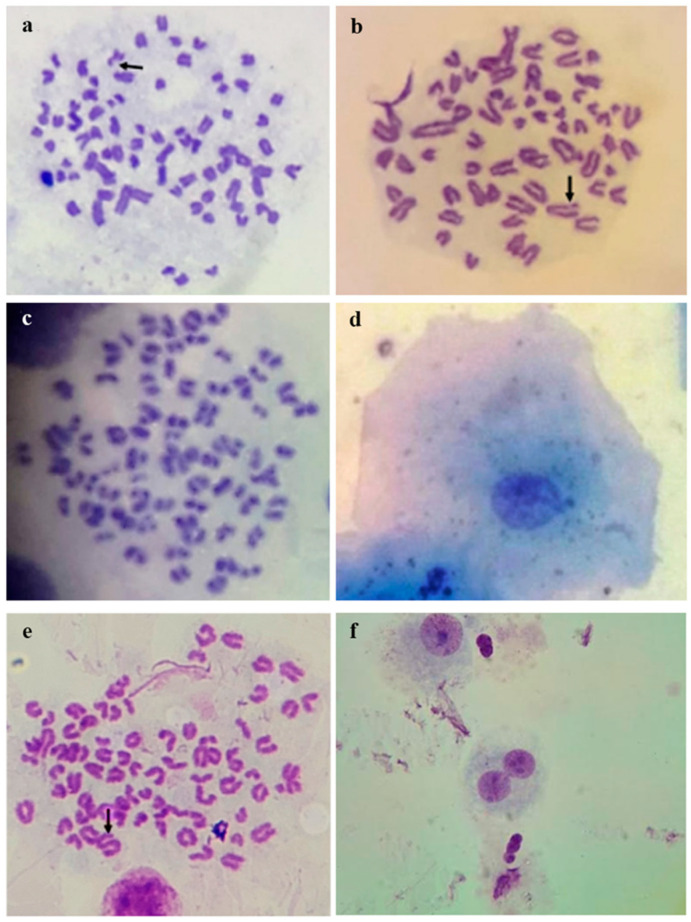
Chromosomal aberrations in dog lymphocytes, Giemsa stained, 2n = 78 [153] (**a**). Chromosome break and chromatid gap; (**b**). Chromatid gap; (**c**). Endoduplication; (**d**). Micronucleus in canine buccal epithelium cell [154]; (**e**). Telomeric association in dog metaphase lymphocyte (Image Caliri M.); (**f**). Binucleated cell and bridge in CBMN micronucleus test in in vitro dog lymphocytes cultures (Image Caliri M.).

Some genotoxicity biomarkers in dog cells are illustrated in Figure 3.

In total, 33 papers were found and reviewed where dogs have been used as health sentinels, through chemical or biological studies, 17 were analyzed for the elaboration of Table 3 under the pre-established selection criteria. A larger number of papers with genotoxicity biomarker studies—specifically chromosomal alterations—has been conducted in dogs in different types of cancers, which validates the use of this biomarker as an indicator of chromosomal instability that may facilitate neoplastic development. Another substantial number of papers report analytical measurements in blood and/or urine of a wide variety of xenobiotics in dogs exposed to environmental contaminants. It is unfortunate that these studies are not accompanied by genotoxic effect biomarkers and are limited to quantifying chemical exposure biomarkers, representing a missed opportunity for an integrated study to address an environmental problem.

The articles detected in the international bibliography where dogs are used in their habitual environments, or canine cells are used to evaluate exposure to chemical and physical agents, are presented in Table 3.

As can be observed in Table 3, the studies found during the web search which were conducted with canine cells using genotoxicity assays, are scarce. For the performance of the in vitro mammalian CA test, the canine karyotype is complex compared to those of other mammals. It has 78 chromosomes, all acrocentric, and of very similar sizes. The TG 473, in its latest update, indicates the analysis of 300 metaphases for searching numerical and structural alterations [20]. Analysis is possible in canines [153], but it is very difficult to obtain 300 metaphases per culture; initially, the requirement was 100 metaphases.

In line with the “One Health” premise and the recommendation of the 3Rs, the best option to evaluate genotoxic risk is the use of animals in their natural environments, such as the ex vivo study by Backer et al. [159], which, as can be inferred from the table, is a less commonly used option. Another ex vivo option is to treat animals in vivo and then obtain blood to reveal possible damage in vitro, as in the study with cadmium by Dönmez-Altuntas et al. [158].

An innovation is the implementation of the micronucleus assay in canine oral epithelial cells (BMCyt). This assay has been used to evaluate countless human groups and has been effectively employed to quantify genetic damage in humans exposed to a wide variety of xenobiotics and situations [32]. The methodological approach of this assay in dogs is a new alternative for evaluating genomic integrity in canines. It has been accompanied by a thorough histological analysis of the buccal area sampled, as corresponds to its application in a new species [154]. Thus, it has potential applications as a biomarker of genotoxicity, both in environmental and clinical studies in dogs.

The ideal approach for sentinel animal studies involves integrating the measurement of contaminants (in tissues/environment) with genotoxicity biomarker evaluation to inform epidemiological research. However, the high cost of analytical chemistry often prevents this comprehensive strategy, particularly in developing nations. Nonetheless, research using non-invasive samples like hair for contaminant analysis in dogs is increasing, as highlighted by recent reviews focusing on POPs, heavy metals, and the “One Health” context [171].

Several studies have confirmed the importance of dogs as bioindicators of environmental quality [37]. They have been used to infer the presence of heavy metals in the environment by quantifying them in ovarian tissue of animals inhabiting contaminated environments [172]; pesticides, polycyclic aromatic hydrocarbons, polychlorinated biphenyls, and polybrominated diphenyl ethers in hair [173] or blood [174]. These types of studies are specific in chemical detection using analytical methods.

Numerous studies associate environmental exposures in dogs with specific cancers: household asbestos and certain flea repellents with asbestos-like fibers with mesothelioma; [175]; urban air pollution with lung cancer/chronic nonspecific pulmonary disease COPD [35]; various pesticides with lymphoma, testicular, and bladder cancer [176,177]; specific PCBs with mammary cancer [178], and components of Agent Orange (Vietnam) with testicular cancer in military dogs [179].

It is economically more accessible to have effective biomarkers, such as genotoxic effect biomarkers, than to perform analytical measurements. Additionally, the canine genome presents greater homology with the human genome than other mammalian model genomes for disease study; even the analysis of the assembly of the complete dog genome sequence shows that it is more homologous in sequence conservation with humans than with mice [180], constituting a shared exposome [36].

Dogs have enzyme and metabolic systems very similar to those of humans, a shorter lifespan, and similar manifestation of certain diseases [181,182]. They are also free from lifestyle-related risk factors for diseases, such as smoking, medication use, and alcohol consumption. Dogs can respond to a toxic agent in a similar way to humans. Epidemiological studies in dogs can be used as a prior alternative, and at a lower cost, compared to epidemiological studies in humans [171]. In summary, dogs can be used as early warning systems to manage actions in public health [37,183].

## 3. In Vivo and In Vitro Tests, Selection of Doses and Concentrations to Be Used

Short-term assays for detecting mutagens and carcinogens began to be developed in the 1970s when the scientific community associated the presence of environmental contaminants with health effects in terms of mutations and cancer development. At that time, the literature examining these early epidemiological associations was extensive, and many of the studies were occupational [184]. Cancer largely develops in the later stages of human life; therefore, laboratory animal tests for newly synthesized chemical compounds or those released by industrial factories involved maintaining animals for their entire lifespan, approximately 2 years in rodents and around 20–30 years in NHPs [185,186]. The duration and high cost of evaluating these suspected carcinogenic agents led the scientific community to design short-term assays, as those presented in this review, for detecting mutagens and carcinogens [187].

A key challenge in genotoxicity testing is extrapolating risks from short-term, high-dose in vitro assays to long-term, low-dose real-world exposures. In vitro assays use high concentrations (often based on cytotoxicity limits) over short durations (e.g., 3–72 h) to elicit detectable damage [17], whereas in vivo assays employ doses based on physiological tolerance (e.g., Maximum Tolerated Dose) administered via relevant exposure routes.

In vitro genotoxicity evaluation should yield results illustrating genotoxicity biomarkers, as well as cytotoxicity, which is typically assessed by the reduction of cell proliferation. The maximum level of cytotoxicity for the acceptance of assays has been more explicitly defined for in vitro cytogenetic assays. It is recommended that if the maximum concentration is based on cytotoxicity, the highest concentration should aim to achieve 55 ± 5% cytotoxicity using the recommended cytotoxicity parameters. For in vivo assays, the maximum dose is usually the highest dose that will be tolerated without evidence of limiting toxicity of the study, such that higher dose levels, based on the same dosing regimen, would be expected to produce lethality or evidence of pain, distress, or suffering that would require humane euthanasia [17].

The usual or most probable route of exposure to the substance being tested will determine the assay to choose. When veterinary drugs used in production animals are evaluated, the most likely route of entry into the population is considered to be oral exposure through residues in food. Conversely, when evaluating air-borne environmental contaminants to which someone may be involuntarily exposed, the dermal or inhalation routes are likely prioritized for assessment. This is very different from intentional exposure, such as the use of drugs or consumption of a certain type of food [188]. If the exposure is massive and intentional, it is likely that a battery of additional tests will be required. If the exposure will only be by susceptible subgroups, it is likely that a minimal battery will be required. Less clear is whether the risk involves a hereditary effect, and if so, an evaluation covering germ cells would most convenient.

Mechanistic studies on the three species reviewed within the framework of genetic toxicology tests are almost non-existent, and there are even fewer comparisons between mammals or to humans. This should be addressed by the scientific community in the future.

## 4. The Present and Projections of Tests for Genetic Toxicology

Genetic toxicology assays were initially used to assess carcinogenic risk, and with the discovery of oncogenes and tumor suppressor genes, it became possible to investigate the contribution of specific mutations to carcinogenesis. Currently, other contributions associated with genomic damage have been added, such as epigenetics, germ cell risk, the accumulation of damage related to aging, and recent advances in OMIC sciences (genomics, proteomics, transcriptomics, epigenomics, lipidomics, and metabolomics, etc.). New Approach Methodologies (NAMs) are described, which are technological, methodological, and combined approaches that can provide information on chemical hazard and risk assessment without the use of animals, including in silico, in chemico, in vitro, and ex vivo approaches [38]. These NAMs are not necessarily newly developed methods, but rather, the substitution of a requirement in conventional assays or how conventional methods are applied in regulatory decision-making [189]. The U.S. Environmental Protection Agency’s plan to end animal testing divides scientists. Some see the future in animal-free models, but others are concerned about the consequences for health.

While human mutagens are typically carcinogens, some carcinogens act via non-mutagenic mechanisms (e.g., chronic inflammation, epigenetic changes, endocrine disruption) [190]. Standard OECD Test Guidelines are designed for hazard identification but offer flexibility; assays can be adapted (e.g., modifying dose, duration, sample timing) to investigate specific modes of action or tissue-specific tumorigenesis.

Modern industrial and technological activities introduce numerous novel chemicals (drugs, pesticides, nanomaterials, organometallic compounds, food additives, and new biomedical materials) into the environment. The variety of new chemical compounds and their high volume resulting from industrial, agricultural and even domestic activities are a source of concern for the scientific community. A second concern is the potential interaction of these agents with DNA and the possibility of causing various alterations (such as point mutations, chromosomal rearrangements, DNA adducts, DNA strand breaks, and an increase in micronuclei). Consequently, evaluating genotoxicity biomarkers is vital for assessing these potential health hazards.

Considerable attention has been devoted by regulatory bodies worldwide to develop objective analyses for the use of mutagenicity test results in regulatory decision-making. With the involvement of the Food and Drug Administration (FDA), the Environmental Protection Agency (EPA), the WHO, the International Programme on Chemical Safety (IPCS), International Cooperation on Harmonisation of Technical Requirements for Registration of Veterinary Medicinal Products (VICH) and the OECD, assurance is provided that in vitro and in vivo methods for detecting the mutagenic and genotoxic potential of newly synthesized substances, as well as those already on the market, are developed, validated, and properly protocoled.

Several international organizations and agencies committed to human and animal health have declared the need for an integrated assessment of human and environmental risks, with the aim of improving risk management and promoting the implementation of public health policies. Evaluating risks in animals naturally exposed to the same contaminants, i.e., estimating the probability that an adverse effect will occur as a result of exposure to a contaminant, offers the possibility of protecting human populations more promptly.

No less important are the epidemiological studies analyzing risk factors associated with exposure to chemicals, and evaluating the health effect biomarkers play, which is an invaluable role. The development and use of genotoxicity biomarkers are rapidly growing to measure specific occupational and environmental exposures, predict the risk of pathological development, or monitor the effectiveness of procedures for controlling exposure to genotoxic chemicals [15].

## 5. Conclusions

This systematic review is focused on three unconventional mammalian models in the “One Health” framework, giving priority to studies that document the evaluation of veterinary and human pharmaceuticals, industrial chemicals as environmental contaminants, and employ tests for genetic toxicology (OECD). Among these, bovines emerge as the strongest model for genotoxicity studies, supported by their large volume of research, the diversity of compounds tested, and the methodological approach encompassing environmental monitoring. In contrast, data on genetic toxicology in NHPs and dogs remain limited, highlighting a gap that warrants more targeted research. If one takes into consideration the most attractive characteristics of both, i.e., the phylogenetic similarity of NHPs to humans and the shared environment with dogs (water, air, and soil components with their potential xenobiotic contaminants) research can further reinforce the “One Health” approach.

Despite the “One Health” framework highlighting sentinel species, many environmental biomonitoring studies still focus on human health extrapolation rather than direct animal impacts. A critical gap remains: the lack of integration between chemical quantification and genotoxicity biomarker evaluation in these sentinels, limiting comprehensive environmental risk assessment.

## Figures and Tables

**Table 1:** Tests for genetic toxicology carried out with non-human primate cells.

OECD Genetic Toxicology Tests	Species	Xenobiotics/ Contaminants	Findings and Conclusions of the Genotoxicological Evaluation	Merits of the Paper	References
**TG 473**	**In vitro mammalian chromosomal aberration (CA) test**				
**and TG 474**	*Macaca mulatta*	Methyl-phenidate hydrochloride	No significant increases in CA frequencies in treated animals. Non-genotoxic or clastogenic effects on cells under the experimental conditions.	Drug commonly used to treat Attention-Deficit/Hyperactivity Disorder in pediatric populations. Drug not classified by IARC. Conflicting results have been reported in relation to Methyl-phenidate genotoxic activity. Peripheral blood lymphocytes cultures were used for testing	[58]
	* Cebus apella, *Macaca fascicularis*, *Erythrocebus patas*	X-ray irradiation	CA increases in a dose-dependent way. Genotoxic effect on NHP’peripheral blood lymphocytes cultures	Ionizing radiation is “carcinogenic to humans” (Group 1 IARC). Species selected due to the chromosomal response is supposed to be similar than human ones.	[59,60]
**TG 474**	**Mammalian erythrocyte micronucleus test**				
	*M. mulatta*	Cyclophosphamide (CP)	Single administration of CP induced a 10-fold increase in blood MN-Ret frequency. Clastogenetic effect on NHP’ peripheral blood lymphocytes culture and bone marrow cells.	Well-established clastogen. Drug “Carcinogenic to humans” (Group 1 IARC). To validate the use of blood lymphocytes instead of the invasive bone marrow sampling.	[61]
	*Callitrhix jacchus*	Methotrexate, CP, Cytosine-rabinoside (Ara-C), 5-fluorouracil	MN erythrocyte frequencies were increased by the Ara-C treatment. This NWM species could serve as a suitable model for testing the genotoxic effects of chemicals.	Four antineoplastic pediatric therapeutic drugs. Methotrexate and 5-fluorouracil are “not classifiable as to their carcinogenicity to humans” (Group 3 IARC). Cyclophosphamide is “carcinogenic to humans” (Group 1 IARC). Ara-C, is not classified by IARC. Blood smears were used for testing.	[62]
**TG 479**	**In vitro sister chromatid exchange assay (SCE) in mammalian cells**				
	*Ateles chamek*, *A. paniscus*	None (healthy NHP)	Spontaneous G-SCEs and T-SCEs were quantified.	The study was designed to validate the use of SCE test for detection of DNA breakage and repair in Neotropical Primates species. Primary cell cultures of fibroblast from both species were used.	[63]
	*Alouatta caraya*, *A. chamek*, *A. paniscus*, ** Sapajus cay	None (healthy NHP)	Spontaneous G-SCEs were quantified.	Four different genome structures were analyzed and compared in the same study. Primary cell cultures of fibroblasts from each species were used.	[64]
	*Homo sapiens*, ** S. cay	Ornidazole (ONZ), Metronidazole (MTZ)	*S. cay* SCE values following treatments were significantly different from control. Genotoxic effect in the assessed conditions.	Two nitro-imidazole antibiotics. Main use: antimicrobial in human and veterinary medicine. MTZ is “possibly carcinogenic to humans” (Group 2B IARC). ONZ is not classified by IARC. Conflicting results have been reported in relation to the genotoxic activity of imidazole derivatives. Peripheral blood lymphocytes culture was used for testing.	[65]
**TG 487 and**	**In vitro mammalian cell micronucleus test and**				
**No TG**	**In vitro mammalian alkaline comet assay**				
	*Cercophitecus aethiops*	Fluconazole	MN frequency increased at 1306 µM of fluconazole, cytotoxic and genotoxic in the assessed conditions.	Triazole antifungal drug. Main use: obstetrics and gynecology for the treatment of vaginal candidiasis, patients with compromised immunity. Not classified by IARC. Scarce studies on the genotoxicity of fluconazole and the need of testing effects in different systems were the driving force of the study. VERO cell line was used for testing.	[66]
	* C. apella	N-Methyl-Nitrosourea (MNU) and Canova complex	MNU significantly increased MN frequency, but declined with Canova. Genotoxic effect of MNU can be controlled by Canova	Synthetic, mutagenic and carcinogenic drug used in research to induce tumors. Possibly carcinogenic to humans (Group 2B IARC). The potential antimutagenic action of Canova, a homeopathic complex of compound was evaluated. Peripheral blood lymphocytes culture was used for testing.	[67]
**No TG**	**In vitro mammalian alkaline comet assay**				
	*C. aethiops*	Dipyrone	Dipyrone causes DNA damage with a dose-response effect. Genotoxic and cytotoxic effects in the assessed conditions.	Common analgesic drug used in human medicine. Not classified by IARC. VERO cell line was used for testing.	[68]
**No TG**	**In vitro mammalian chromosomal aberration test with fragile sites induction**				
	*A. caraya*, *Saimiri* *boliviensis*	Fluorodeoxyuridine	Low coincidence between c-fra location, and breakpoints involved in rearrangements.	DNA synthesis inhibitor. Not classified by IARC. Common fragile sites are reliable markers of genetic instability, useful to evaluate the spontaneous change in chromosome structure. Peripheral blood lymphocytes cultures were used.	[69]
**No TG**	**Comet chip assay in Primary macaque hepatocytes (PMHs)**				
	*M. mulatta*	Enzymes, sugars, hormones, anti-inflammatories, corticosteroids, and others (22 compounds)	Primary macaque hepatocytes constitute a reliable surrogate of primary human’s hepatocytes for evaluating the genotoxic hazards of chemical substances.	In vivo and in vitro study/ Validation of a non-human primate putative surrogate for primary human hepatocytes in genotoxicity assessments for compounds known to have different genotoxic/carcinogenic effects.	[70]
**No TG**	**In vivo and ex vivo Peripheral blood cell with the γ-H2AX assay**				
	*M. mulatta*	^137^Cs γ-rays	Quantitation of γ-H2AX foci is a good bio-dosimeter for analyzing body exposure to radiation.	Gamma-radiation is “Carcinogenic to humans” (Group 1 IARC). Study performed to validate the γ-H2AX biodosimetry for estimating severity and chronicity of therapeutic body radiation exposure effects. Peripheral blood lymphocytes and hair bulbs were tested.	[71]

* *C. apella* = *Sapajus apella*. ** Previously *C. cay = Cebus libididinosus* = *C. apella paraguayanus* = *C. apella*. IARC: International Agency for Research on Cancer. Agents classified by the IARC Monographs, Volumes 1–138 (https://monographs.iarc.who.int/list-of-classifications) accessed on 20 January 2025; Report of the Advisory Group to Recommend Priorities for the IARC Monographs during 2020–2024 (https://monographs.iarc.who.int/wp-content/uploads/2019/10/IARCMonographs-AGReport-Priorities_2020-2024.pdf) accessed on 20 January 2025.

**Table 2:** Genotoxicity studies of pesticides and veterinary drugs in cattle during 2005–2025.

OECD Genetic Toxicology Tests	Xenobiotics	Findings and Conclusions of the Genotoxicological Evaluation	Merits of the Paper	References
**TG 473**	**In vitro mammalian chromosomal aberration (CA) test ^a^**			
	Acetamiprid-Mospilan 20SP®	Decrease of MI and a high percentage of ruptures was observed (25 and 50 μg/mL). Possible genotoxic action on chromosomes	Commercial formulation. Neonicotinoid pesticide (main use: insecticide). Not classified by IARC	[104]
	Thiacloprid-Calypso 480 SC®	Increase of Cas (240 and 480 ug/mL). Reduction in the MI. Genotoxic action on chromosomes	Commercial formulation. See Thiacloprid’s merits of the paper above	[105]
	Mitomycin C	Increase CAs and decrease of MI. Structural CAs (FISH-WCP). Genotoxic ability	Alkylating agent used in veterinary medicine. Other uses in genotoxicity studies. Possibly carcinogenic to humans (Group 2B IARC)	[106]
	Tolylfluanid-Euparen Multi®	Decrease of MI. Dose-dependent polyploidy and non-reciprocal translocations chromosomes 5 and 7 (FISH-WCP). Cytostatic and cytotoxic capacity	Commercial formulation. Sulfamides pesticide (fungicide) use in agriuclture. Not classified by IARC	[107]
	Tolylfluanid-Euparen Multi®-Bendiocarbamate	Decrease of MI (160 µg/mL bendiocarb). Dose-dependence polyploidy (FISH-WCP). Cytotoxic capacity	Commercial formulations (Tolylfluanid) for use in agriculture. See Tolylfluanid’s merits of the paper above. Bendiocarbamate is a insecticide used in agriculture and in indoor areas. Not classified by IARC	[108]
	Glyphosate–Monsanto Europe®	Increase of polyploidy (4n) (56 umol/L) (FISH-WCP). Potential genotoxic effect (aneugenic)	Commercial formulation. Phosphanate herbicide. Probably carcinogenic to humans (Group 2A IARC)	[109]
	Glyphosate–Monsanto Europe®	Increase non-significant of monosomy, trisomy, and polyploidy (4n) (FISH-WCP). Genotoxic ability	Commercial formulation. See Glyphosate’s merits of the paper above	[110]
	Tolylfluanid–Euparen Multi®, Bendiocarbamate	Inhibition of MI, polyploidy, chromosomes 1 and 5 translocations, (FISH-WCP). Tolylfluanid caused numerical CAs. Genotoxic capacity (tolyfluanid)	Commercial formulation. See merits of the paper of Tolylfluanid and Bendiocarbamate above	[111]
and TG 479	Prothioconazole + tebuconazole- Bayer®	MI Reduction (dose-dependent 7.5–30 μg/mL). Breakages and polyploidy (3 μg/mL) (FISH-WCP). Decreased the PI. No significant increase of SCE. Cytotoxic capacity on lymphocytes	Commercial formulation. Mixture. Both are triazoles pesticides (fungicides), main use in agriculture, other human and veterinary medicine. Not classified by IARC	[112]
	Tolylfluanid–Euparen Multi®	No evidence on the clastogenicity. Increases of chromosomal damage (17.5 μg/mL). Cytostatic ability	Commercial formulation. See Tolylfluanid’s merits of the paper above	[113]
	Glyphosate–Monsanto Europe®	Decrease of MI (560 and 1120 mmol/L). Increase non-significant of chromatid breaks and gaps. Increase in SCE (24 h exposure). Further increase in S9lymphocytes (2 h at 140 mmol/L). Genotoxicity and cytostatic ability	Commercial formulation. See Glyphosate’s merits of the paper above	[114]
and TG 479, TG 487	Epoxiconazole	Increase of CAs (2.5 and 50 μg/mL). Decrease of MI (100 μg/mL) and PI (24 h at 100 μg/mL and 48 h lower exposures). Dose-dependent decrease of CBPI. Cytostatic and cytotoxic capacity on lymphocytes	Trizole pesticide (fungicide), main use in agriculture, other human and veterinary medicine. Not classified by IARC	[115]
	Tebuconazole–Orius 25 EW®	Increase of CAs (6–30 µg/mL). Numerical CAs chromosomes 5 and 7 (FISH-WCP). Increase SCE (24 h at 15–60 µg/mL). Dose-dependent decrease of CBPI. Cytotoxic ability and possible genotoxic (clastogenic) effect.	Commercial formulation. See Tebuconazole’s merits of the paper above	[116]
and TG 479, TG 487, SCGE in vitro assay (No TG)	Thiacloprid	Elevation of CAs (120 μg/mL). Elevations in SCEs (120–480 μg/mL). Decrease of CBPI 30–480 μg/mL. DNA damage (240–480 µg/mL). Cytostatic and cytotoxic ability in bovine blood cells	Neonicotinoid pesticide (main use: insecticide). Not classified by IARC	[117]
	Epoxiconazole + fenpropimorph- Tango Super®	Decrease in MI and PI (3–15 µg/mL). Decrease in CBPI (1.5–15 µg/mL). Moderate elevation in DNA damage non significance. Cytostatic and cytotoxic capacity on lymphocytes	Commercial formulation Mixture. See Epoxiconazole ’s merits of the paper above. Fenpropimorph is a morpholine-derived pesticide (fungicide) use in agriculture. Not classified by IARC	[118]
	Thiacloprid	Increased breakage rate (120, 240, 480 µg/mL). Non-significant increase in CAs (FISH-WCP). Decrease in PI (48 h at 240 and 480 µg/mL). Decrease in the CBPI. Increases in lymphocyte DNA damage (120 and 480 μg/mL). Genotoxic and cytotoxic ability in lymphocytes	See Thiacloprid’s merits of the paper above	[119]
**TG 479**	**In vitro Sister Chromatid Exchange Assay in Mammalian Cells (SCE) ^a^**			
	Tolylfluanid-Bayer®	Increased genetic damage (17.5 µg/mL). Decrease of PI (3.5–17.5 µg/mL). Genotoxic capacity	Commercial formulation. See Tolylfluanid’s merits of the paper above	[120]
	Tebuconazole-Orius®	Elevations in the mean of SCEs at 24 h exposure. Genotoxic and cytotoxic capacity	Commercial formulation. See Tebuconazole’s merits of the paper above	[121]
**TG 487**	**In vitro mammalian cell micronucleus test (CBMN assay) ^a^**			
	Cypermethrin (Cyp), chlorpyrifos (Cpf) and mixture Cyp+Cpf	Cyp produced a decrease in CBPI and an increase in BNMN and BNBuds. Cyp and Cyp+Cpf increased BNMN. Genotoxic (Cyp and Cpf) and cytoxic (Cyp) capacity	Pesticides used in agriculture and like parasicticides in veterynary medicine. Cyp is a insecticide pyrethroid and Cpf is an organophosphate insecticide and acaricide. Both not classified by IARC. Cyp has high priority to be evaluated by IARC	[122]
and SCGE in vitro assay (no TG)	Doramectin-Dectomax-sf®	Increased BNMN and NBuds in lymphocytes and cumulus cells, (40–60 ng/mL). Increased proportion of damaged lymphocyte nuclei. Genotoxic and cytotoxic capacity	Commercial formulation. Avermectin class used for the treatment of parasites in animals. Not classified by IARC.	[123]
	Thiacloprid-Calypso®	Decrease of CBPI (30–240 μg/mL). Dose-dependent increase of BNMN (120 and 240 μg/mL). Induction of DNA- dsb in lymphocytes using neutral single-cell microgel. Potential genotoxic and cytotoxic capacity	Commercial formulation. See Thiacloprid’s merits of the paper above	[124]
	Enrofloxacin-Floxagen®	Increase of BNMN in lymphocytes independent of the concentration and increased of NBuds. Dose-dependent increase of ID in lymphocytes (50, 100 and 150 μg/mL). Genotoxic and cytotoxic capacity	Commercial formulation. Quinolinemonocarboxylic acid (Quinolone) used like veterinary antibacterial agent. Not classified by IARC.	[125]
	Tebuconazole+ prothioconazole-Prosaro 250EC®	Dose-dependent decrease in the CBPI at 48 h. Dose-dependent increase in DNA damage. Genotoxic and cytotoxic capacity	Commercial formulation. Mixture. See merits of the paper of Tebuconazole and Prothioconazole above	[126]
**TG 474**	**Mammalian in vivo Erythrocyte Micronucleus Test**			
	BCG vaccine recombinant *Mycobacterium bovis*	3 vaccines with different *M. bovis* proteins cause MNE, although these decrease over time. Genotoxic and possible cytotoxic capacity (myelosuppression)	Not classified by IARC.	[127]
**TG 489**	**In Vivo Mammalian Alkaline Comet Assay (SCGE assay) ^a^**			
and CBMN ex vivo assay (No TG)	Glyphosate-Roundup®	Glyphosate feed did not induce DNA damage, did not induce genotoxic effects. No evidence of genotoxic capacity	Commercial formulation. See Glyphosate’s merits of the paper above	[128]
**No TG**	**Ex vivo mammalian cell micronucleus test (CBMN assay) ^a^**			
	Cypermethrin+ chlorpyrifos-Ecto 2A Plus®	Positive correlation between BNMN and BNBud post dermal exposure to a therapeutic dose. No evidence of genotoxic capacity	Commercial formulation. Mixture. See merits of the paper of Cypermethrin and Chlorpyrifos above	[103]

^a^ All assays were performed on lymphocytes, with exceptions that are mentioned. Results *p* ≤ 0.05 are presented, with exceptions clarified. CAs: chromosome aberration, SCGE: single-cell gel electrophoresis; CBMN: Cytokinesis-block micronucleus cytome assay, MI: mitotic index, BNMN: binucleated cells with micronuclei, NPBs: nucleoplasmic bridges, NBuds: nuclear buds, CBPI: nuclear proliferation index, ID: index damage, FISH-WCP: fluorescent in situ hybridization analysis with whole chromosome painting probes, PI: proliferation index, MNE: Micronucleated normochromatic erythrocyte; DNA-dsb: DNA double-strand breaks. IARC: International Agency for Research on Cancer. Agents classified by the IARC Monographs, Volumes 1–138 (https://monographs.iarc.who.int/list-of-classifications) accessed on 20 January 2025; Report of the Advisory Group to Recommend Priorities for the IARC Monographs during 2020–2024 (https://monographs.iarc.who.int/wp-content/uploads/2019/10/IARCMonographs-AGReport-Priorities_2020-2024.pdf) accessed on 20 January 2025.

**Table 3:** Genotoxicity studies carried out with dog cells.

OECD Genetic Toxicology Tests	Xenobiotic/ Contaminant	Findings and Conclusions of the Genotoxicological Evaluation	Merits of the Paper	References
**TG 473**		**In vitro mammalian chromosomal aberration (CA) test**		
	Mytomicin C (MMC)	CA were significantly higher due to the effect of Mitomycin C (*p* = 0.0247).	MMC is one of the drugs recommended by the OECD for the CA assay. Given the scarce use of dog lymphocytes in this assay, it is the first step to be performed.	[153]
**TG 474**		**Mammalian erythrocyte micronucleus (MN) test**		
	Cyclophosphamide (CP) and etoposide (ETP)	Dose-related MN responses were evident for both agents.	In vivo study/5 days CP and 2 days ETP treatments. Support for utility of flow cytometry-based blood and bone marrow MN-retyculocytes (RET) measurements in dogs. Both are chemotherapeutics and “Carcinogenic to humans” (Group 1 IARC).	[155]
	CP	The kinetics of appearance of MN in blood maximum frequency occurred ~48 h after dosing.	In vivo study/One therapeutic dose of CP, The three-color flow cytometric method uses anti-CD71 labeling to identify reticulocytes.	[156]
	None (Healthy dogs)	Quantification of spontaneous values	In vivo study/Blood smears, acridine orange 100x objective- This species could be used as monitors for genotoxic events.	[157]
**TG 487**		**In vitro mammalian cell micronucleus test (CBMN in lymphocyte cultures)**		
	Cadmium (Cd)	Cd might be directly and/or indirectly genotoxic after a monthly oral administration in dogs.	Occupational exposure to Cd is associated with cancers of the lung. Ex vivo study/orall adminstration. Priority to be classified by IARC.	[158]
	Superfund sites	Pet dogs living near the Superfund sites had a higher micronucleus frequency than control animals	In situ exposure/ex vivo test Area contaminated with high levels of organochlorine pesticides, DDE, DDT, lindane; volatile organic chemicals.	[159]
	X-irradiation and 3-aminobenzamide (3 AB)	Canine lymphocytes have been found to be about three times more radiosensitive than human lymphocyte	Ionizing radiation is “carcinogenic to humans” (Group 1 IARC). In vitro, 3 AB inhibits poly(ADP-ribose) polymerase activity and increase thegenotoxic effect of X-rays	[160]
	Military working dogs	Increase in chromosomal damage.	In situ exposure/ex vivo test Animals assessed before and 6 months after deployment	[161]
**No TG**		**In vivo mammalian cell micronucleus cytome test (BMNCyt)**		
	None (Healthy dogs)	Dogs presented genetic damagemarkers similar to those in humans but at a different frequency.	Support the potential development of BMCyt in buccal epithelial cells in dogs as biomarkers of disease	[162]
	None (Healthy dogs)	A significant increase of micronuclei, nuclear buds and total nuclear aberrations frequencies in purebred dogs compared to mixed-bred dogs	The observed increased genomic damage amongst purebred dogs may not be due to a different genomicinstability typical of a particular breed, but to inbreeding itself.	[163]
	Stress	Evidence of a possible correlation between physiological stress conditions and higher levels of genomic damage in sheltered dogs	Priority to be classified by IARC (job stress)	[164]
	Piperazine	BMCyt/in administered puppies, karyolitic cells were observed to be twice as frequent following treatment (*p* < 0.05)	The animals were studied in the first usual deworming scheme for newborn.	[165]
**No TG**		**Mammalian Alkaline Comet Assay (** **Single Cell Gel Electrophoresis, SCGE assay)**		
	Silver nanoparticles	increase in micronuclei, nuclear buds and nucleoplasmic bridges	In vitro peripheral blood. Priority to be classified by IARC	[166]
	Household cigarette smoke	Statistically significant differences were found between exposedand non-exposed to cigarette smoke in comet assays carried out on biopsy samples not in swab samples.	In situ exposure/ex vivo test Priority to be classified by IARC (second nand smoke). More than 40 mutagenic and carcinogenic agents present in cigarette smoke.	[167]
	Air particulate matter index (PM10)	Alkaline comet length from olfactory or respiratory epithelia of dogs were 67.86 and 72.46, fespectively. Comet length increases with dog age	In situ exposure/in vivo study in olfactory and respiratory epithelia. Priority to be classified by IARC. Air pollution, as measured by PM10, can be responsible for this DNA damage.	[168]
	Nutritionally levels of selenium	U-shaped dose-response between selenium and DNA damage within the prostate	In vivo/Study lasting 7 months. To determine the optimal intake of selenium for prostate cancer prevention. Priority to be classified by IARC	[169]
**No TG**		**In vitro and in vivo Peripheral Blood Mononuclear Cell with the γ-H2AX assay**		
	Metro- nidazole (MTZ)	MTZ 36 μg/m serum without henotoxic effect. In vitro, MTZ led to a significant increase in DNA damage at 100 μg/mL	In vitro and in vivo studies comparison. In vivo MTZ dosages for 1 week. MTZ is “possibly carcinogenic to humans”. H2AX histone phosphorylation in the cellular response against DNA double-strand breaks.	[170]

DDT: dichlorodiphenyltrichloroethane. DDE: dichlorodiphenyldichloroethylene. IARC: International Agency for Research on Cancer. Agents classified by the IARC Monographs, Volumes 1–138 (https://monographs.iarc.who.int/list-of-classifications) accessed on 20 January 2025; Report of the Advisory Group to Recommend Priorities for the IARC Monographs during 2020–2024 (https://monographs.iarc.who.int/wp-content/uploads/2019/10/IARCMonographs-AGReport-Priorities_2020-2024.pdf) accessed on 20 January 2025.

## Data Availability

Not applicable.

## List of Acronyms/Abbreviations

BMCyt	Buccal Micronucleus Cytome assay
BNMN	Binucleated cells with micronuclei
CA	Chromosomal aberrations
CBMN	Cytokinesis Block Micronucleus assay
CIN	Chromosomal instability
COPD	Chronic nonspecific pulmonary disease
CP	Cyclophosphamide
CPF	Chlorpyrifos
CYP	Cypermethrin
DL-PCBs	Dioxin-like polychloro biphenyls
DL-PCDFs	Dioxin-like polychloro dibenzofurans
DNA-dsb	DNA double-strand breaks
DSB	Double strand breaks
EFSA	European Food Safety Authority
EPA	Environmental Protection Agency
FDA	Food and Drug Administration
FISH	Fluorescence in situ hybridization
ID	Index damage
IPCS	International Programme on Chemical Safety
MI	Mitotic index
MN	Micronuclei
MNE	Micronucleated normochromatic erythrocyte
MNU	N-Methyl Nitrosourea
MRLs	Maximum Residue Limits
MTZ	Metronidazole
NAMs	New Approach Methodologies
NBuds	Nuclear buds
NHPs	Non-human primates
NPBs	Nucleoplasmic bridges
NWM	New World Monkeys
OECD	Organization for Economic Cooperation and Development
ONZ	Ornidazole
OWM	Old World Monkeys
PCBs	Polychlorinated Biphenyls
PCDDs	Polychlorodibenzodioxins
PI	Proliferation Index
Ret	Reticulocytes
SCE	Sister Chromatid Exchange
SCGE	Single Cell Gel Electrophoresis
TG	Test Guideline for Genetic Toxicology
UDS	Unscheduled DNA Synthesis
VICH	International Cooperation on Harmonisation of Technical Requirements for Registration of Veterinary Medicinal Products
WCP	Whole Chromosome Painting Probes
